# Effect of exogenous γ-aminobutyric acid on physiological property, antioxidant activity, and cadmium uptake of quinoa seedlings under cadmium stress

**DOI:** 10.1042/BSR20240215

**Published:** 2024-06-26

**Authors:** Xiao Hua Hao, Ke Xin Liu, Meng Yuan Zhang

**Affiliations:** Department of Biology, Xinzhou Teachers University, Xinzhou, Shanxi 034000, China

**Keywords:** γ-aminobutyric acid, antioxidant system, cadmium(Cd) stress, physiological characteristics, quinoa

## Abstract

Increasing cadmium (Cd) pollution has negative effects on quinoa growth and production. Gamma-aminobutyric acid (GABA) confers plants with stress resistance to heavy metals; however, the mechanism remains unclear. We explored the effects of exogenous GABA on the physiological characteristics, antioxidant capacity, and Cd accumulation of quinoa seedlings under Cd stress using hydroponic experiments. Partial least-squares regression was used to identify key physical and chemical indices of seedlings affecting Cd accumulation. Compared with those of the CK group, exposure to 10 and 25 µmol·L^−1^ Cd significantly reduced the photosynthetic pigment contents, photosynthesis, and biomass accumulation of quinoa seedlings; resulted in shorter and thicker roots; decreased the length of the lateral roots; decreased the activities of superoxide dismutase (SOD) and peroxide (POD); and increased H_2_O_2_ and malondialdehyde (MDA) contents. Exogenous GABA reduced the Cd content in the stem/leaves and roots of quinoa seedlings under Cd stress by 13.22–21.63% and 7.92–28.32%, decreased Cd accumulation by 5.37–6.71% and 1.91–4.09%, decreased the H_2_O_2_ content by 38.21–47.46% and 45.81–55.73%, and decreased the MDA content by 37.65–48.12% and 29.87–32.51%, respectively. GABA addition increased the SOD and POD activities in the roots by 2.78–5.61% and 13.81–18.33%, respectively, under Cd stress. Thus, exogenous GABA can reduce the content and accumulation of Cd in quinoa seedlings by improving the photosynthetic characteristics and antioxidant enzyme activity and reducing the degree of lipid peroxidation in the cell membrane to alleviate the toxic effect of Cd stress on seedling growth.

## Introduction

With development of the metallurgy industry, the long-term inappropriate use of cadmium (Cd)-based agricultural chemicals, and sewage irrigation, the soil Cd pollution level in China has reached a severe and critical state. Cd is a non-essential element of plants and is highly toxic. Cd present in the soil is easily absorbed by crops and subsequently enters the human body through the food chain, thus threatening human health [1]. Previous studies have shown that a high concentration of Cd can cause a series of physiological toxic reactions in plants to inhibit photosynthesis and chlorophyll synthesis [2], interfere with the energy supply and metabolic processes, destroy the intracellular redox homeostasis, cause oxidative damage, disturb normal cell structure and function, and finally inhibit plant growth and development, leading to leaf yellowing, overall biomass reduction, and even death [3].

Previous studies demonstrated that exposure to high Cd stress severely inhibited the growth of quinoa seedlings, and the photosynthetic parameters, carotenoids, root fresh weight, and soluble protein content were significantly reduced, while the contents of malondialdehyde (MDA) and soluble sugars increased with the increase of Cd concentration [4,5]. As a complete protein alkaline food source that is rich in amino acids, quinoa is considered to have the highest nutritional value among all traditional food crops; thus, quinoa is considered to be the only crop food that can meet all of the nutrient requirements of the human body. However, relative to maize and rice, quinoa has greater potential for Cd accumulation in the grains, which is mainly due to its stronger ability to transport Cd from the roots to the stem and leaves. Therefore, alleviating the effect of Cd stress on the growth of quinoa and improving the ability of quinoa to resist Cd stress have become important goals [6]. At present, the main methods to improve the resistance of quinoa to Cd stress include agronomic measures such as water management or application of conditioning agents, genetic breeding methods such as screening of quinoa varieties with Cd tolerance or low Cd accumulation, and the application of exogenous substances to improve the Cd resistance of the seedlings, which mainly include chemical substances given their high efficiency and low cost.

Exogenous plant growth regulators can effectively alleviate the damage of abiotic stress on plants. γ-Aminobutyric acid (GABA) is a four-carbon non-protein amino acid that exists in a free state in various organisms, which plays an important role in plant growth and development and stress resistance [7,8]. GABA is an essential non-protein amino acid. Many studies have shown that GABA accumulates in large quantities when plants are subjected to abiotic stresses, and acts as a signaling molecule to regulate tolerance to various abiotic stresses [9]. Under stress conditions, exogenous GABA can reduce the damage caused by the production of reactive oxygen species (ROS) via improving the activity of protective enzymes in plants, and can further improve the stress resistance of crops by helping to regulate the intracellular osmotic pressure and photosynthesis. However, the application of GABA as an exogenous substance to improve the Cd resistance of quinoa is rare in practice.

Therefore, the aim of this study was to evaluate the effects of GABA on the growth characteristics, photosynthetic characteristics, antioxidant enzyme activity, and Cd accumulation of quinoa seedlings under Cd stress using a hydroponic experimental setup. We further established a partial least-squares (PLS) regression model to determine the key factors affecting Cd accumulation in seedlings. Overall, these findings can provide a more comprehensive understanding of the mechanism by which GABA alleviates Cd toxicity in quinoa seedlings, offering a theoretical basis and technical reference for the application of GABA in reducing the toxic effects of Cd in economically important crops.

## Methods

### Seedling culture and treatment

The seed of quinoa tested was white quinoa (JQ00304, purchased from Shuozhou City, Shanxi Province). Studies shown that the content of GABA in this quinoa is low [10], which can reduce the experimental error in this experiment.

The source of Cd and GABA are cadmium chloride (CdCl_2_) and GABA (CAS 56-12-2, Aladdin), and the nutrient solution is Hoagland nutrient solution.

Quinoa seeds with full particles and uniform size were selected and first treated with 5% hydrogen peroxide (H_2_O_2_). After 15 min of disinfection, the seeds were rinsed with deionized water four to five times, incubated in seedling trays at 25°C for 24 h, and transferred to the greenhouse for culture. When the seeds sprouted and reached a length of approximately 1 cm, the seedlings were transferred to the plant light incubator. After growing to the one-leaf stage, the consistently growing seedlings were transplanted into a hydroponic basin containing 2 L of Holland (Hoagland) nutrient solution. To facilitate adaptation of the quinoa seedlings to the hydroponic environment, they were cultured with a 1/8 concentration of nutrient solution after transplanting, and then the concentration was gradually increased to 1/4, 1/2, and complete successively. During this period, the nutrient solution was replaced every 3 days, and the seedlings were subjected to the experimental treatments when they had developed two leaves. Five experimental treatment groups were established: 0 µmol·L^−1^ Cd + 0 mg·L^−1^ GABA (control group [CK], with only nutrient solution added), 10 µmol·L^−1^ Cd + 0 mg·L^−1^ GABA (Cd_10_ group), 25 µmol·L^−1^ Cd + 0 mg·L^−1^ GABA (Cd_25_ group), 10 µmol·L^−1^ Cd + 100 μmol·L^−1^ GABA (Cd_10_+GABA group) and 25 µmol·L^−1^ Cd + 100 μmol·L^−1^ GABA (Cd_25_+GAGA group). Three replicates were set for each treatment. Cadmium chloride (CdCl_2_) was used as the Cd source in all treatments.

The relative humidity of the artificial climate chamber was maintained at 60–65%, the photoperiod was set to 16 h light/8 h dark under a day/night temperature of 25°C/20°C, and the light intensity was 400 μmol·(m^2^·s)^-1^.

### Determination of physiological and biochemical indicators

Seedling samples were collected at 14 days of treatment and the shoot and undershoots of the seedlings were isolated. Before sampling, the plant photosynthetic parameters were measured using a portable photosynthetic meter (LI-6400, LICOR Inc., U.S.A.). The samples were soaked in a 20 mmol·L^−1^ Na_2_-EDTA solution for 15 min, washed with deionized water four to five times, and a portion of the fresh sample was placed in an envelope, heated in an oven for 30 min at 105°C, and then dried at low temperature (70°C) to a constant weight to determine the biomass. The remaining portions of the samples were crushed in liquid nitrogen and stored at −80°C until the measurement of physiological and biochemical indicators.

The leaf gas parameters, including the net photosynthetic rate (*P*_n_), transpiration rate (*T*_r_), intercellular CO_2_ concentration (*C*_i_), and stomatal conductivity (*G*_s_), were measured on the first fully expanded leaf formed on the top of the quinoa plant, with measurements taken from three plants per treatment. The light intensity was controlled at 1000 µmol·(m^2^·s)^−1^ and the temperature was controlled at 25°C. Total root length, root surface area, root volume, mean root diameter, and total root tips were measured by a root scanner system (V700 PHOTO, Epson, Japan). The leaf chlorophyll content was determined by 95% ethanol extraction. The MDA content was determined by the thiobarbituric acid method. H_2_O_2_ levels were determined by the titanium sulfate color method. Superoxide dismutase (SOD) activity was measured by the nitrogen blue tetrazolium reduction method and peroxidase (POD) activity was measured according to the guaiacol method.

### Measurement of Cd content

After drying and crushing, the plants were digested with a nitric acid:perchloric acid solution at a 3:1 volume ratio and the Cd content was determined with an atomic absorption spectrometer (PinAAcle 900T, U.S.A.). To ensure the reliability of the experimental data, the Cd content was simultaneously measured using the national standard plant sample GBW07604 (GSV-1) for quality control. The recovery rate of the spike was between 87% and 113%.

### Data analysis

To evaluate the influence of the phenotypic traits and physiological and biochemical indices of quinoa on Cd accumulation, we established separate regression models for above-ground tissue Cd accumulation, under-ground Cd accumulation, and total Cd accumulation, and the key factors affecting Cd accumulation were determined according to the dimensionless variable importance projection (VIP) value in the PLS regression equation. The PLS model integrates correlation analysis, multiple linear regression analysis, and principal component analysis, which can not only effectively address the problem of multi-collinearity among independent variables but also effectively reduces the dimensions in factor analysis to reveal the key factors affecting the dependent variables among multi-dimensional independent variables [11]. The VIP value can intuitively and quantitatively reflect the importance of the respective variables in predicting the dependent variable. The tolerance index reflects the degree of inhibition of plant growth in stress environments, which is calculated according to the following equation:

$$\text{Tolerance index}\;=\;\frac{\text{dry weight}}{\;\text{dry weight of control group}}\times 100\%$$

We also calculated the root-crown ratio and transport coefficient with the following equations:

$$\text{Root crown ratio}\;=\;\frac{\text{under-ground tissue dry weight}}{\text{above-ground tissue dry weight}}$$

Statistical analysis of the data was performed using Microsoft Excel 2016 and SPSS statistical software version 27.0. Using the minimum significant extreme range method (least-significant difference, *P*<0.05), data were compared among groups by analysis of variance. PLS regression was carried out with SIMCA-P 11.5 for key factor selection. Data were visualized using Origin Pro 2018 and Adobe Illustrator 2019 software [12].

$$\text{Transport coefficient}\;=\;\frac{\text{Stem-leaf Cd accumulation}}{\text{root Cd accumulation}}\times 100\%$$

## Results

### Effect of exogenous GABA on the biomass and tolerance of quinoa seedlings under Cd stress

As shown in Table 1, compared with those of the control (CK), the root length, stem length, root dry weight, and stem and leaf dry weights of quinoa seedlings under the Cd_10_ treatment significantly decreased by 45.32%, 18.17%, 49.04%, and 63.36%, respectively. Under Cd_25_ treatment, the root length, stem length, root dry weight, and stem and leaf dry weight of quinoa seedlings significantly decreased by 64.07%, 33.37%, 57.69%, and 67.27%, respectively, compared with those of the CK group. These results demonstrated that Cd stress significantly inhibited the normal growth of quinoa seedlings in a concentration-dependent manner. Moreover, compared with that of the CK, the root-to-shoot ratio of the seedlings under Cd stress increased to different degrees, indicating that the inhibition effect of Cd on the above-ground parts of quinoa seedlings was greater than that on the roots. The tolerance index of the stems and leaves was also lower than that of the seedling roots, further indicating that stems and leaves were more sensitive to Cd stress. In addition, the tolerance indices of both the above-ground and under-ground parts were lower under Cd_25_ treatment than under Cd_10_ treatment.

**Table 1:** Effects of exogenous GABA application on the biomass and tolerance index of quinoa seedlings under cadmium (Cd) stress

Group	Root length (cm)	Stem leaf length (cm)	Root dry weight (g.plant^−1^)	Stem and leaf weight (g.plant^−1^)	Root-to-shoot ratio	Root tolerance index (%)	Stem/leaf tolerance index (%)
CK	47.71 ± 5.12a	28.68 ± 5.44a	0.104 ± 0.007a	0.333 ± 0.007a	0.31 ± 0.02bc	100.00 ± 6.24a	100.00 ± 2.23a
Cd_10_	26.09 ± 1.74b	23.47 ± 0.25bc	0.053 ± 0.011bc	0.122 ± 0.017bc	0.43 ± 0.05ab	50.96 ± 10.77bc	36.64 ± 5.35b
Cd_25_	17.14 ± 1.55c	19.11 ± 2.10cd	0.044 ± 0.002c	0.109 ± 0.017bc	0.40 ± 0.07ab	42.31 ± 2.43c	32.73 ± 5.46b
Cd_10_+GABA	28.41 ± 1.31b	25.43 ± 2.33ab	0.060 ± 0.002b	0.131 ± 0.015b	0.45 ± 0.09a	57.70 ± 2.37b	39.33 ± 4.93b
Cd_25_+GABA	25.55 ± 3.21b	24.58 ± 1.63ab	0.062 ± 0.012b	0.131 ± 0.021b	0.47 ± 0.09a	59.62 ± 13.11b	39.34 ± 6.38b

Note: Data are presented as mean ± standard deviation. Different lowercase letters in the same column indicate significant differences between different treatments (*P*<0.05).

Table 1 further shows that exogenous GABA application promoted the growth of quinoa seedlings under Cd stress to varying degrees. In the Cd_10_+GABA group, the root length, stem length, root dry weight, and stem and leaf dry weight of quinoa seedlings were increased compared with those under treatment of Cd_10_ alone, although the differences were not statistically significant and these indices remained lower than those of the CK group. In the Cd_25_+GABA treatment, the root length, stem length, root dry weight, and stem and leaf dry weight of the quinoa seedlings increased compared with those of the Cd_25_ group, with significant increases found in all cases except for the stem and leaf dry weight; however, all of these values were still lower than those of the control. With respect to the tolerance index, the Cd_10_+GABA treatment inhibited the weight of the roots and the weight of the stems and leaves by 42.30% and 60.67%, respectively, compared with those of the control group; however, the inhibition was greater with the Cd_10_ treatment alone (49.04% and 63.36%, respectively). The Cd_25_+GABA treatment group showed inhibition in the weight of the roots and the weight of the stems and leaves by 40.38% and 60.66%, respectively, compared with those of the control, which were again reduced compared with the inhibition found for the Cd_25_ treatment alone (57.69% and 67.27%, respectively). These results indicated that exogenous GABA could alleviate the inhibition effect of Cd stress on the growth of quinoa seedlings to a certain extent.

### Effect of exogenous GABA on leaf photosynthetic parameters and chlorophyll content of quinoa seedlings under Cd stress

As shown in Table 2, compared with those of the control (CK), the photosynthetic parameters of seedling leaves under the Cd_10_ and Cd_25_ treatments were significantly decreased. Specifically, the Pn, Gs, Ci, and Tr of quinoa seedlings decreased by 36.49%, 64.00%, 15.96%, and 55.76%, respectively, under the Cd_10_ treatment and by 44.98%, 68.00%, 41.92%, and 59.68%, respectively, under the Cd_25_ treatment. These results indicated that the photosynthesis of quinoa seedling leaves was inhibited to a certain extent under Cd stress, with a greater inhibitory effect under high Cd stress levels.

**Table 2:** Effect of exogenous γ-aminobutyric acid (GABA) on photosynthetic parameters and chlorophyll content of quinoa seedlings under cadmium (Cd) stress

handle	Pn (use CO_2_ count)/ μ mol · (m^2^·s)^-1^	Gs (in H_2_O) /mol·(m^2^·s)^-1^	Ci (in CO_2_) / μ mol·mol^−1^	Tr (in H_2_O) /mmol·(m^2^·s)^-1^	ω (Chlorophyll a) /mg·g^−1^	ω (Chlorophyll b) /mg·g^−1^	ω (Carotenoids) /mg·g^−1^	ω [Chlorophyll (a + b)] /mg·g^−1^
CK	30.75 ± 0.77a	0.50 ± 0.14a	459.13 ± 28.35a	8.16 ± 1.77a	1.867 ± 0.003a	0.657 ± 0.001a	0.308 ± 0.001a	2.530 ± 0.003a
Cd_10_	19.53 ± 4.63bc	0.18 ± 0.06bc	385.87 ± 26.65b	3.61 ± 1.21bc	1.039 ± 0.001d	0.438 ± 0.000d	0.156 ± 0.000c	1.473 ± 0.001d
Cd_25_	16.92 ± 4.73c	0.16 ± 0.07bc	266.67 ± 38.38c	3.29 ± 1.33bc	0.922 ± 0.023e	0.361 ± 0.013e	0.152 ± 0.002c	1.234 ± 0.037e
Cd_10_+GABA	24.56 ± 4.09ab	0.21 ± 0.05b	447.01 ± 25.98a	4.77 ± 0.95b	1.594 ± 0.003b	0.609 ± 0.001b	0.247 ± 0.001b	2.213 ± 0.02b
Cd_25_+GABA	23.67 ± 5.01b	0.20 ± 0.08b	420.00 ± 28.98ab	4.38 ± 1.42b	1.123 ± 0.000c	0.558 ± 0.001c	0.152 ± 0.000c	1.618 ± 0.001c

Note: Data are presented as mean ± standard deviation. Different lowercase letters in the same column indicate significant differences between different treatments (*P*<0.05).

Photosynthetic parameters can reflect the strength of photosynthesis [13]. Table 2 shows that compared with those in the single Cd_10_ treatment group, the Pn, Gs, Ci, and Tr of quinoa seedling leaves in the Cd_10_+GABA group increased by 25.76%, 16.67%, 15.84%, and 32.13%, respectively, although these parameters were still lower than those of the control and the differences were not statistically significant except for the increase in Ci. Similarly, compared with those in the single Cd_25_ treatment group, Pn, Gs, Ci, and Tr of quinoa seedlings under the Cd_25_+GABA treatment were increased by 39.89%, 25.00%, 57.50%, and 33.13%, respectively, but were still lower than those in the CK. These results indicated that exogenous GABA treatment could alleviate the photosynthetic inhibition of quinoa seedlings under Cd stress to a certain extent.

Photosynthetic pigments play important roles in the absorption and transmission of light energy in plant photosynthesis. The contents of photosynthetic pigments in plant leaves also reflect the degree of external stress [14]. As shown in Table 2, compared with those of the CK group, the contents of chlorophyll a, chlorophyll b, carotenoids, and total chlorophyll in the leaves of quinoa seedlings significantly decreased under Cd stress alone, with a decrease of 44.35%, 33.33%, 49.35%, and 41.79%, respectively, in the C_10_ group, and of 50.62%, 45.05%, 50.65%, and 51.23%, respectively, in the C_25_ group. After exogenous GABA supplementation, the photosynthetic pigment content in the leaves of seedlings was significantly increased compared with that measured under single Cd stress (*P*<0.05), but was still significantly lower than that under the control condition. The contents of chlorophyll a, chlorophyll b, carotenoid, and total chlorophyll in the leaves of quinoa seedlings treated with Cd_10_+GABA were increased by 53.42%, 39.04%, 58.33%, and 50.24%, respectively, compared with those treated with Cd_10_ alone. Compared with Cd_25_+GABA treatment, the contents of chlorophyll a, chlorophyll b, carotenoid, and total chlorophyll in the leaves of quinoa seedlings were increased by 21.80%, 54.57%, 0.08%, and 31.12%, respectively. The above results showed that Cd stress inhibited and destroyed the synthesis and accumulation of chlorophyll in quinoa seedling leaves, resulting in varying degrees of chlorophyll content reduction in a Cd concentration-dependent manner. Exogenous GABA can alleviate the damage of Cd stress on the photosynthesis of quinoa seedlings by increasing the content of photosynthetic pigments in the leaves.

### Effect of exogenous GABA on the root morphology of quinoa seedlings under Cd stress

The root system has the ability to absorb and transport water, nutrients, and mineral elements [15,16]. The root morphological indices of quinoa seedlings under different treatments are shown in Table 3. Overall, compared with the CK condition, the root length, root surface area, root volume, and total tip number of quinoa seedlings under the Cd_10_ treatment significantly decreased by 69.47%, 65.73%, 60.47%, and 70.87%, respectively, and the average diameter increased by 12.02%. Under the Cd_25_ treatment, the root length, root surface area, root volume, and total tip number of quinoa seedlings significantly decreased by 82.78%, 78.53%, 72.31%, and 87.07%, respectively, and the average diameter increased by 26.74%. These results showed that Cd stress could inhibit the normal root growth of quinoa seedlings, resulting in a shorter root length, fewer lateral roots, and thicker roots, and the Cd_25_ treatment had a stronger inhibitory effect on the root growth of quinoa seedlings than the Cd_10_ treatment.

**Table 3:** Effects of exogenous GABA on quinoa root morphology under cadmium (Cd) stress

Treatment	Root length/cm	Root surface area/cm^2^	Root volume/cm^3^	Average root diameter/mm	Total root tip number
CK	1755.55 ± 136.35a	143.07 ± 7.74a	0.903 ± 0.027a	0.258 ± 0.005c	3394 ± 300.63a
Cd_10_	535.97 ± 88.11b	49.03 ± 8.88bc	0.357 ± 0.077c	0.289 ± 0.011b	988.76 ± 453.31b
Cd_25_	302.26 ± 20.08c	30.72 ± 0.95c	0.250 ± 0.011cd	0.327 ± 0.015a	438.87 ± 89.23c
Cd_10_+GABA	606.06 ± 173.88b	64.25 ± 20.36b	0.546 ± 0.188b	0.318 ± 0.010a	768.01 ± 151.14bc
Cd_25_+GABA	450.29 ± 59.02bc	44.93 ± 4.33bc	0.356 ± 0.022c	0.0335 ± 0.011a	592.07 ± 53.50bc

Note: Data are presented as mean ± standard deviation. Different lowercase letters in the same column indicate significant differences between different treatments (*P*<0.05).

As shown in Table 3, compared with those of the Cd_25_ treatment, the root length, root surface area, root volume, and total tip number of quinoa seedlings under the Cd_25_+GABA treatment were increased by 48.97%, 46.26%, 42.40%, and 39.91%, respectively, although the differences were not statistically significant and the levels were still lower than those of the CK. Compared with those of the Cd_10_ treatment, the root length, root surface area, and root volume of quinoa seedlings in the Cd_10_+GABA treatment were increased by 13.08%, 31.04%, and 52.94%, respectively, with a significant difference found for the root volume, although the levels were still lower than those of the CK. These results indicated that exogenous GABA promoted the water and nutrient absorption of seedling roots by expanding the root surface area and increasing the root volume, and thus improved the Cd resistance of quinoa seedlings.

### Effects of exogenous GABA on H_2_O_2_ and MDA contents of quinoa seedlings under Cd stress

**Figure 1:**
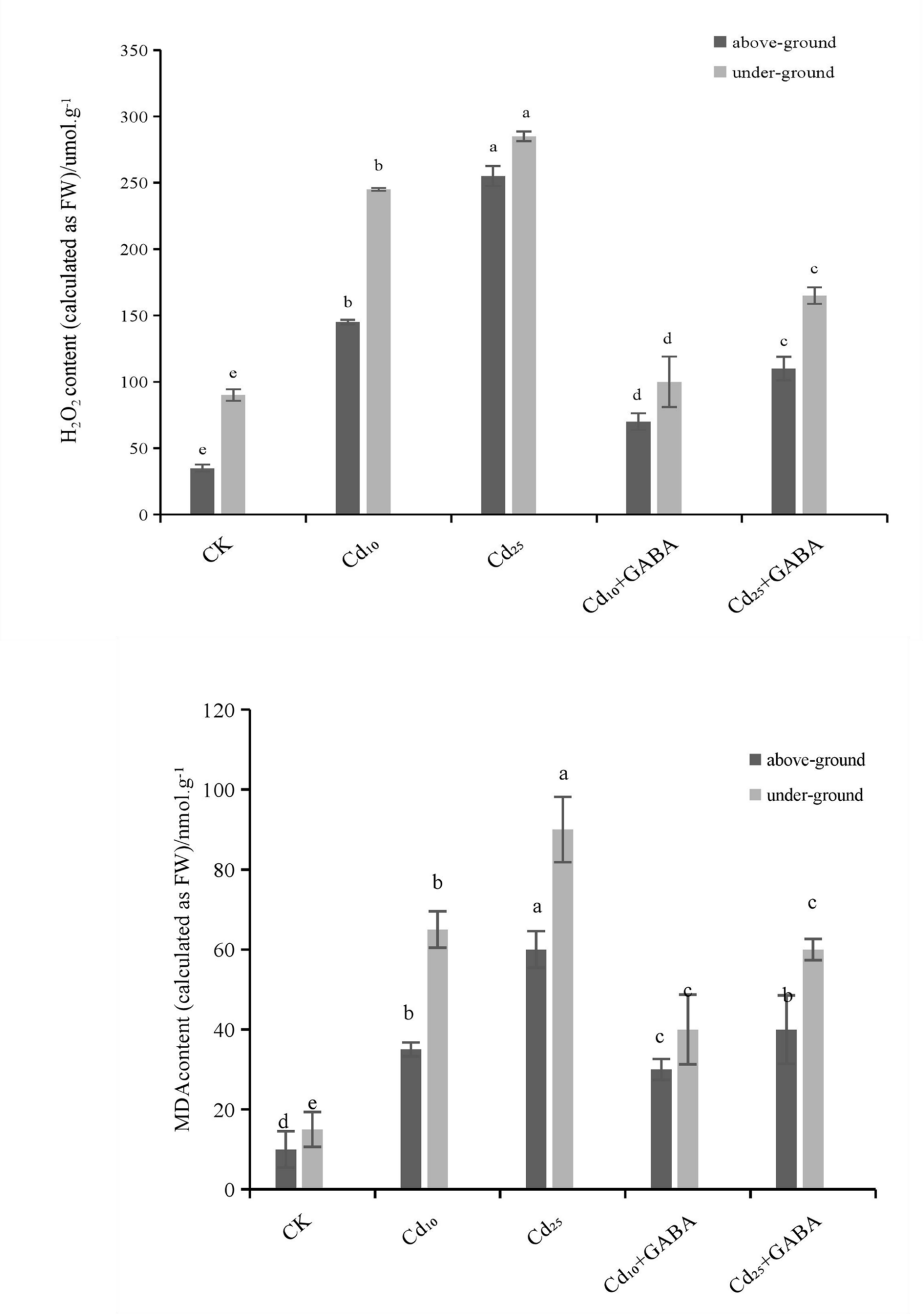
Effects of exogenous GABA on the H_2_O_2_ and MDA contents of quinoa seedlings under cadmium (Cd) stress Different lowercase letters indicate a significant difference among the five treatments (*P*<0.05).

To clarify the mitigation effect of exogenous GABA on cell oxidative damage in quinoa seedlings under Cd stress, the H_2_O_2_ and MDA contents in the shoots and under-ground parts of the seedlings were determined. As shown in Figure 1, when compared with those of the CK, the H_2_O_2_ and MDA contents in the roots and leaves of quinoa seedlings were significantly increased under single Cd_10_ and Cd_25_ stress (*P*<0.05), and the increase rate enhanced with the increase of Cd concentration. The H_2_O_2_ and MDA contents in the roots of quinoa seedlings under the Cd_10_ treatment were 2.37 and 10.34 times higher than those of the CK, and those in the leaves were 4.48 and 2.26 times higher than those of the control, respectively. However, compared with those in the Cd_10_ treatment, the H_2_O_2_ and MDA contents in the roots and leaves of quinoa seedlings treated with Cd_10_+GABA were significantly decreased (*P*<0.05). The contents of H_2_O_2_ and MDA in the roots decreased by 47.46% and 48.12%, and those in the leaves decreased by 45.81% and 29.87%, respectively. Under high-concentration Cd stress (Cd_25_), the H_2_O_2_ and MDA contents in the roots of quinoa seedlings were 3.20 and 11.52 times those of the control, and the H_2_O_2_ and MDA contents in the leaves were 7.79 and 3.25 times those of the control, respectively. However, compared with those in the Cd_25_ treatment, H_2_O_2_ and MDA contents in the roots and leaves of quinoa seedlings treated with Cd_25_+GABA were significantly decreased (*P*<0.05), with a decrease of 38.21% and 37.65% in the roots and a decrease of 55.73% and 32.51% in the leaves, respectively. The above results showed that Cd stress can induce oxidative damage in quinoa seedlings, with a greater effect at higher Cd concentrations, whereas GABA helps to alleviate the oxidative damage induced by Cd and thus the toxicity of Cd to the seedlings.

### Effect of exogenous GABA on the antioxidant enzyme activity in quinoa seedlings under Cd stress

**Figure 2:**
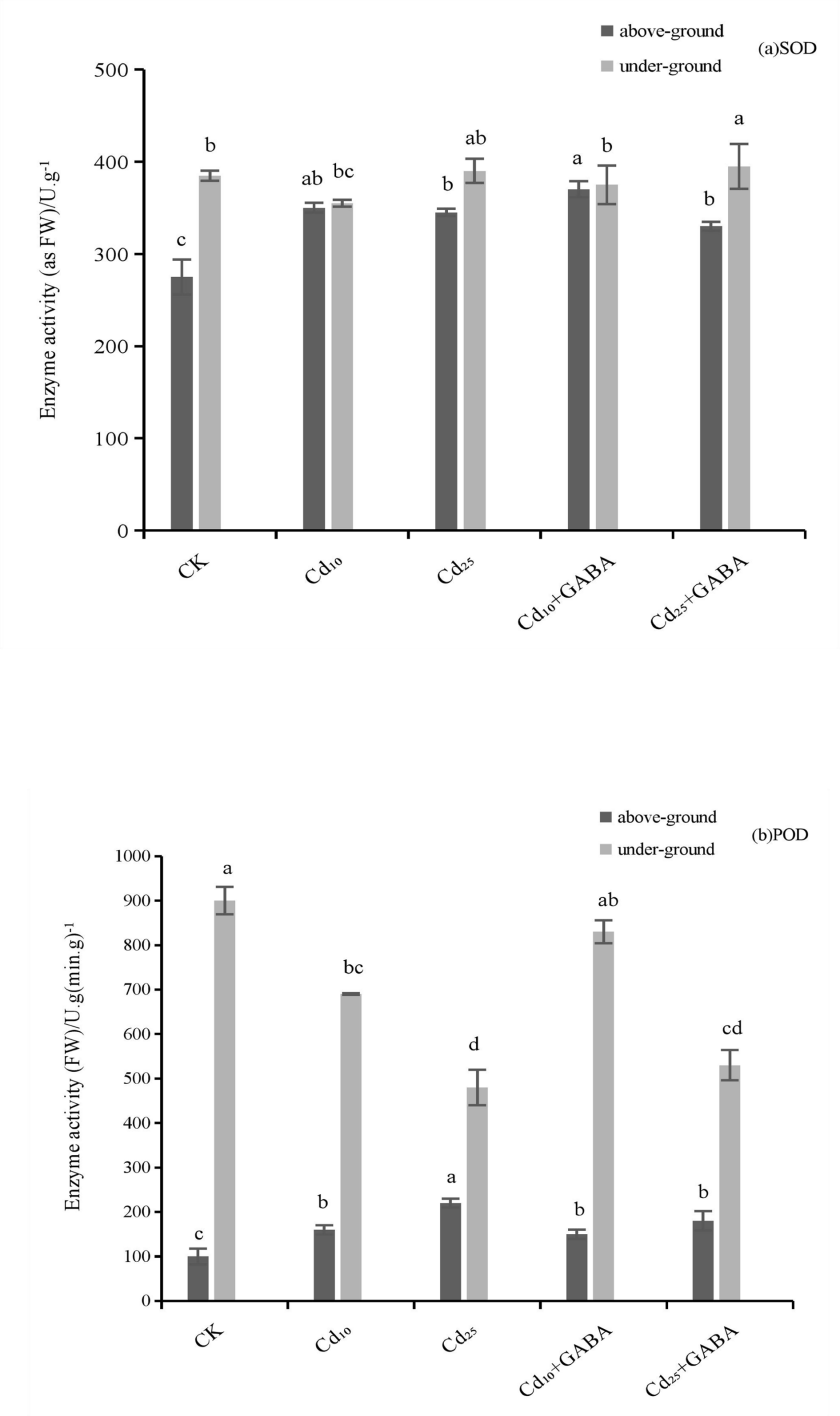
Effect of exogenous GABA on the activities of the antioxidant enzymes (**A**) SOD and (**B**) POD in quinoa seedlings under Cd stress. Different lowercase letters indicate a significant difference between treatments (*P*<0.05).

Figure 2 shows the effects of GABA on the activities of antioxidant enzymes (SOD and POD) in the roots and leaves of quinoa seedlings under Cd stress. Compared with those in the CK, the activities of SOD and POD in quinoa seedling leaves were significantly increased. The POD activity in seedling roots significantly decreased under Cd_10_ and Cd_25_ stress alone (*P*<0.05), whereas Cd stress had no significant effect on SOD activity in the seedling roots. The SOD and POD activities in the Cd_10_ group increased by 24.05% and 50.23%, respectively, in the leaves of quinoa seedlings and decreased by 4.10% and 19.61%, respectively, in the roots, compared with those in the control. There was no difference in the SOD and POD activities in the roots and leaves of quinoa seedlings between the Cd_10_ and Cd_10_+GABA groups. Compared with those of the control, the SOD activity in the roots and leaves increased by 2.78% and 4.11%, respectively; POD activity in the leaves decreased by 1.46% and increased by 18.33% in the roots. The SOD and POD activities in the leaves of quinoa seedlings of the Cd_25_ group were increased by 21.08% and 89.24%, respectively; the POD activity in the roots was decreased by 43.65%, whereas the SOD activity in the roots was not significantly different from that of the control. There were no significant differences in the SOD and POD activities in the roots or stems and leaves of seedlings between the Cd_25_ and Cd_25_+GABA groups, except for a significant reduction in POD activity in the leaves and stems in the Cd_25_+GABA group. SOD and POD activities in the leaves decreased by 2.24% and 11.52%, respectively, whereas those in the roots increased by 5.61% and 13.81%, respectively.

### Effect of exogenous GABA on the Cd content and transport coefficient in quinoa seedlings under Cd stress

Table 4 shows the Cd content in different tissues of quinoa seedlings under different treatments. In both the shoots and roots, the Cd content was significantly higher in the Cd_25_ group than in the Cd_10_ group, indicating that Cd accumulation in quinoa seedlings increased with increasing Cd concentration. Specifically, the Cd content in the roots under the Cd_10_ and Cd_25_ treatment was 11.18 and 11.47 times higher than that of the stems and leaves, with total Cd accumulation increasing by 4.72 and 4.52 times, respectively, indicating that the majority of Cd absorbed by quinoa seedlings accumulated in the roots. When Cd_10_ and GABA were added together, the Cd content significantly decreased in both the roots and in the stems and leaves of quinoa seedlings (by 17.92% and 13.22%, respectively) compared with that of the Cd_10_ alone group. The addition of GABA decreased Cd accumulation in the roots and stems/leaves by 4.09% and 6.71%, respectively, and the transport coefficient decreased by 3.69%, although the difference was not statistically significant. The Cd content in the roots and stems/leaves of quinoa seedlings under Cd_25_+GABA treatment was significantly decreased compared with that under single Cd_25_ treatment (by 28.32% and 21.63%, respectively). Cd accumulation in the roots and stems/leaves decreased by 1.91% and 5.37%, respectively, and the transport coefficient was decreased by 2.76%, with no significant difference. These results indicated that exogenous GABA could alleviate Cd uptake in quinoa seedlings to some extent and also reduce Cd transport from the roots to the stems and leaves.

**Table 4:** Effect of exogenous GABA on cadmium (Cd) content, accumulation, and transport coefficient in quinoa seedlings under Cd stress

Treatment	Under-ground parts		Above-ground parts		Transport coefficient
	ω (Cd) (dry weight) /µg·g^−1^	Accumulation of Cd /µg·plant^−1^	ω (Cd) (dry weight) /µg·g^−1^	Accumulation of Cd /µg·plant^−1^	
CK	–	–	–	–	–
Cd10	1021.56 ± 0.39b	64.77 ± 10.51a	124.55 ± 0.77c	14.08 ± 1.72ab	0.212 ± 0.013a
Cd25	1829.35 ± 0.27a	79.18 ± 2.63a	170.13 ± 1.26a	18.25 ± 3.58a	0.225 ± 0.041a
Cd10+GABA	1174.93 ± 34.45c	59.28 ± 3.78a	101.67 ± 1.09d	13.64 ± 2.09b	0.205 ± 0.019a
Cd25+GABA	1285.05 ± 23.84b	72.33 ± 6.15a	132.48 ± 3.94b	15.35 ± 0.87ab	0.219 ± 0.024a

Note: Data are presented as mean ± standard deviation.‘–’ is not detected. Different lowercase letters in the same column indicate a significant difference among the five treatments (*P*<0.05).

### Determination of key factors affecting Cd accumulation in quinoa seedlings

**Figure 3:**
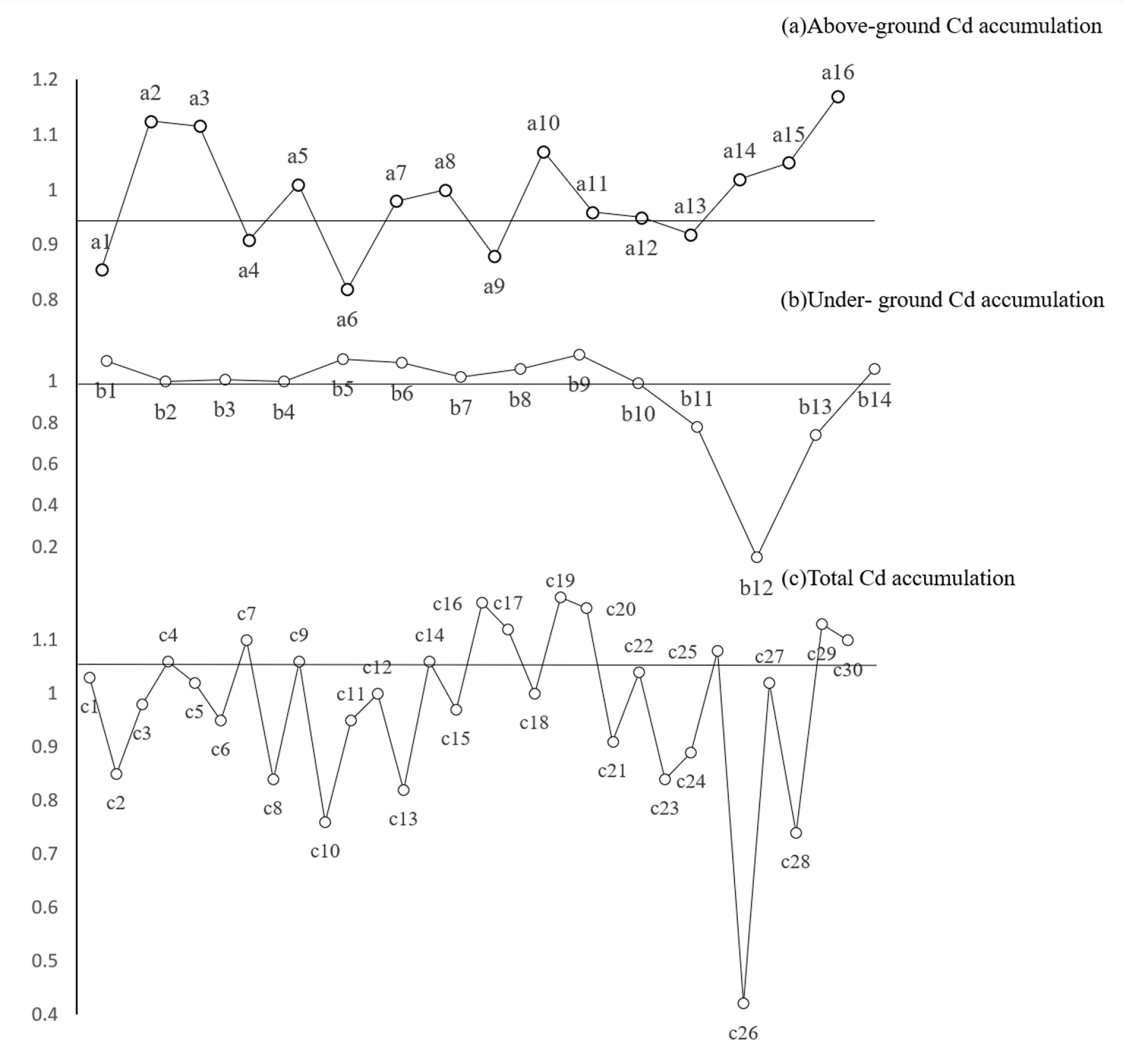
Analysis of factors influencing cadmium accumulation in quinoa seedlings based on the PLS regression model a1, c2: stem-leaf length; a2, c4: stem and leaf dry weight; a3, c7: stem-leaf tolerance index; a4, c8: net photosynthetic rate; a5, c9: stomatal conductance; a6, c10: intercellular CO_2_ concentration; a7, c11: transpiration rate; a8, c12: chlorophyll a; a9, c13: chlorophyll b; a10, c14: carotenoids; a11, c15: chlorophyll (a + b); a12, c21: above-ground MDA content; a13, c23: above-ground H_2_O_2_ content; a14, c25: above-ground SOD activity; a15, c27: above-ground POD activity; a16, c29: above-ground Cd content; b1, c1: root length; b2, c3: root dry weight; b3, c5: root-crown ratio; b4, c6: root tolerance index; b5, c16: total root length; b6, c17: root surface area; b7, c18: root volume; b8, c19: average root diameter; b9, c20: total root tip number; b10, c22: under-ground MDA content; b11, c24: under-ground H_2_O_2_ content; b12, c26: SOD activity in under-ground tissues; b13, c28: POD activity in under-ground tissues; b14, c30: Cd content in under-ground tissues.

Based on the VIP values of the PLS model [17,18], we determined the relative influence of phenotypic traits and physiological and biochemical indices of quinoa seedlings on the total, above-ground tissue, and under-ground tissue Cd accumulation (Figure 3). A larger VIP value indicates a stronger influence and greater ability of the indicator in predicting the change of the dependent variable. Among the phenotypic traits and physiological and biochemical indicators of the above-ground tissues of quinoa seedlings, those with a VIP value greater than 1.0 were the above-ground Cd content, stem and leaf dry weight, stem and leaf tolerance coefficient, carotenoid content, and above-ground POD activity. The above-ground Cd content was determined to be the most critical factor in the response to changes in above-ground Cd accumulation, followed by stem and leaf dry weight and stem and leaf tolerance index. In the under-ground tissues of the seedlings, the parameters with VIP values greater than 1.0 were the total number of root tips, total root length, root surface area, under-ground Cd content, average root diameter, root volume, and root-to-shoot ratio. The total number of root tips was the most critical factor identified in response to the change of Cd accumulation in the under-ground parts, followed by the total root length and root surface area. For the overall seedlings, the parameters with a VIP value greater than 1.0 were the mean root diameter, total number of root tips, total root length, above-ground Cd content, stem and leaf tolerance coefficient, stem and leaf dry weight, root surface area, under-ground Cd content, root length, above-ground SOD activity, carotenoid content, and above-ground POD activity. The average root diameter was the most important factor identified in the response to Cd accumulation, followed by the number of root tips and root length.

**Figure 4:**
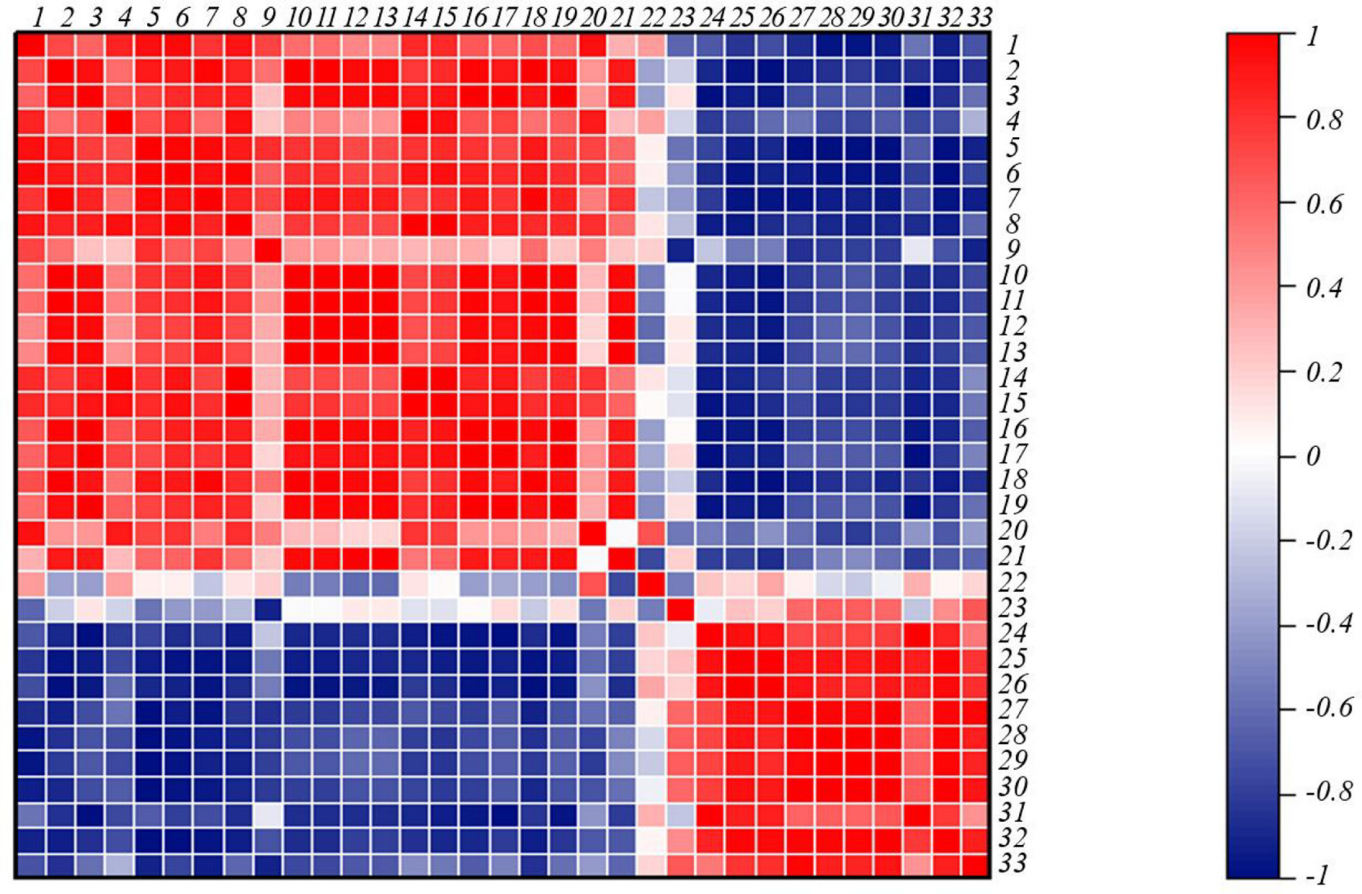
Correlation analysis of the changes in each indicator in response to exogenous GABA treatment of quinoa seedlings under Cd stress 1. Above-ground SOD activity. 2. Root-shoot ratio 3. Under-ground SOD activity. 4. Root mean diameter. 5. Above-ground Cd accumulation. 6. Above -ground Cd content. 7. Total Cd accumulation. 8. Under-ground Cd accumulation. 9.Under-ground Cd content. 10. Above-ground MDA content. 11. Above-ground H_2_O_2_ content. 12. Under-ground H_2_O_2_ content. 13. Under-ground MDA content. 14. Above-ground POD activity. 15. Chlorophyll b. 16. Stem and leaf length. 17. Intercellular CO_2_ concentration. 18. Under-ground POD activity. 19. Root dry weight. 20. Root tolerance index. 21.Transpiration rate. 22. Net photosynthetic rate. 23. Chlorophyll a. 24. Chlorophyll (a+b). 25. Carotenoid. 26.Total root length. 27. Root surface area. 28.Root length. 29. Root volume. 30. Stem and leaf dry weight. 31. Stem and leaf tolerance index. 32. Stomatal conductance. 33. Total number of root tips.

Correlation analysis (Figure 4) showed that the root length, stem leaf length, rot dry weight, stem weight, Pn, Gs, Tr, chlorophyll a, carotenoid, total chlorophyll, root surface area, root volume, and total root tip number had significant negative correlations with Cd content and Cd accumulation in both the above- and below-ground parts (*P*<0.01), whereas the mean root diameter, MDA content, and H_2_O_2_ content showed significant positive correlations with Cd content and Cd accumulation in the above- and under-ground parts (*P*<0.01).

## Discussion

### Exogenous GABA promotes the growth of quinoa seedlings under Cd stress

Cd stress can lead to plant growth and development abnormalities, hinder the plant’s photosynthetic capacity and chlorophyll synthesis, interfere with water and nutrient absorption and transport, cause oxidative damage, and disrupt normal cell structure and function [19,20]. Ultimately, these effects result in a decrease of biomass, yield, and quality of the plants. In the present study, the plant height, root length, and dry matter mass of the roots, stems, and leaves of quinoa seedlings were significantly reduced under 10 and 25 µmol· L^−1^ Cd stress, and the growth of quinoa seedlings was significantly inhibited in a Cd concentration-dependent manner. In addition, the root-to-shoot ratio of the seedlings under Cd stress increased to varying degrees. This may indicate that the stems and leaves of quinoa seedlings are more sensitive to Cd stress. The correlation analysis showed that the root length, stem and leaf length, root dry weight, and stem and leaf dry weight of quinoa seedlings were significantly negatively correlated with the Cd content and Cd accumulation in both the above-ground and under-ground parts (*P*<0.01), which further confirmed that high-concentration Cd stress could lead to Cd toxicity and inhibit the normal growth of quinoa seedlings.

Previous studies have shown that exogenous GABA, as a plant anti-stress inducer, can improve the resistance of plants to heavy metal stress and alleviate the damage of heavy metals to plants [21]. We confirmed that exogenous GABA can promote the growth of quinoa seedlings under Cd stress. The root length, stem length, root dry weight, stem and leaf dry weight, and tolerance index of quinoa seedlings treated with Cd_10_+GABA and Cd_25_+GABA were increased to varying degrees compared with those measured under the corresponding single Cd stress treatment, indicating that exogenous GABA can alleviate the inhibition effect of Cd stress on the growth of quinoa seedlings to a certain extent. The root length, root surface area, root volume, and total tip number of quinoa seedlings under the Cd_10_ and Cd_25_ treatments were significantly reduced compared with those of the CK, whereas the average root diameter was significantly increased. Therefore, 10 and 25 µmol·L^−1^ Cd stress significantly inhibited the root growth and development of quinoa seedlings, showing a trend of shorter root length, fewer lateral roots, and thicker roots. This effect is attributed to the fact that the proteins in quinoa roots can chelate and fix Cd^2+^, thus, when the concentration of Cd^2+^ reaches a certain threshold, the root cells will be accelerated toward lignification, resulting in shorter and thicker roots. The correlation analysis showed that the Cd content and Cd accumulation in the above-ground and under-ground parts of the seedlings were significantly negatively correlated with root surface area, root volume, and total number of root tips (*P*<0.01), but were significantly positively correlated with the average root diameter (*P*<0.01). These results further confirmed that high-concentration Cd stress would lead to shorter and thicker roots and reduce the lateral roots of quinoa. The root length, root surface area, and root volume of quinoa seedlings treated with Cd_10_+GABA and Cd_25_+GABA were increased to varying degrees compared with those of the corresponding single Cd stress treatments.

### Exogenous GABA enhances the antioxidant capacity of quinoa

Cd stress can induce the production and accumulation of ROS in plants, which leads to a disorder of cell metabolism and damages the normal growth and metabolism of plants [22]. H_2_O_2_ is the main ROS that causes the oxidative damage induced by Cd stress, and its abundant production in plant cells will aggravate membrane lipid peroxidation and have a toxic effect on cells [23]. MDA is a product of lipid peroxidation in cell membranes, and its content is commonly used to assess the level of membrane peroxidation and the degree of damage [24]. In this study, we found that the H_2_O_2_ and MDA contents in the roots and leaves of quinoa seedlings were significantly increased under Cd_10_ and Cd_25_ stress in a Cd concentration-dependent manner (Figure 1, *P*<0.05), indicating that both the roots and leaf cell membranes of quinoa seedlings were severely damaged by oxidation with greater toxic damage caused by high-concentration Cd stress. The H_2_O_2_ and MDA contents in the roots and leaves of quinoa seedlings were significantly decreased after exogenous GABA was added compared with those under the single Cd stress condition (*P*<0.05). Therefore, exogenous GABA can help to alleviate the damage to the cell membrane of quinoa seedlings under Cd stress, demonstrating that the antioxidant oxidase system is an important ROS clearance system in plants.

SOD can dismutate superoxide anions to produce H_2_O_2_ and O_2_, while POD can transform H_2_O_2_ into H_2_O, thereby reducing ROS and alleviating the membrane damage caused by membrane lipid peroxidation. Under Cd_10_ and Cd_25_ stress, SOD and POD activities in the leaves of quinoa seedlings increased significantly; however, in the roots, the POD activity decreased significantly and there was no change in SOD activity. This indicates that, on the one hand, high concentration of Cd stress inhibited enzyme activity and metabolic pathways in the roots, and the ability of the roots to clear ROS decreased [25,26]; on the other hand, the stems and leaves of seedlings can reduce the oxidative damage of Cd stress by increasing the antioxidant enzyme activity. After exogenous GABA was added, SOD and POD activities in the roots and leaves of quinoa seedlings increased to varying degrees compared with those measured under the single Cd stress condition, suggesting that exogenous GABA could alleviate the chemical damage of quinoa seedlings under Cd stress by improving antioxidant oxidase activity.

### Exogenous GABA inhibits the absorption and accumulation of Cd by quinoa

The Cd content in the roots of quinoa seedlings under the Cd_10_ and Cd_25_ treatment was 11.18 and 11.47 times that in the stems and leaves, respectively; similarly, the Cd accumulation in the roots under the Cd_10_ and Cd_25_ treatment was 4.72 and 4.52 times that in the stems and leaves, respectively. Thus, the Cd content and Cd accumulation in the under-ground and above-ground parts of quinoa seedlings under CD_25_ treatment were higher than those under Cd_10_ treatment to varying degrees. PLS model analysis showed that the total number of root tips was the key factor affecting Cd accumulation in the under-ground parts of quinoa seedlings, and the mean root diameter was the key factor affecting Cd accumulation in the seedlings.

The roots are the main organs for plants to absorb water and nutrients, and are thus the first tissues to sense and respond to external stress. The correlation analysis showed that the Cd content and Cd accumulation in the above-ground and under-ground parts of the seedlings were significantly negatively correlated with root surface area, root volume, and total number of root tips (*P*<0.01), and were significantly positively correlated with mean root diameter, MDA content, and H_2_O_2_ content (*P*<0.01). After exogenous GABA was added, the Cd content in the roots, stems, and leaves of quinoa seedlings was significantly lower than that in the Cd single stress group. In addition, the Cd accumulation and transport coefficient of the seedlings decreased, suggesting that exogenous GABA could alleviate the absorption and transport of Cd in quinoa seedlings to a certain extent. The low accumulation of Cd in plants is closely related to a series of complex physiological processes. However, the detailed mechanism by which GABA reduces the absorption and transport of heavy metals, especially Cd, in quinoa remains unclear, necessitating further studies by molecular biological approaches such as identifying the changes in key functional genes/proteins in plants [27,28].

## Data Availability

Data and materials are available upon request.

## Abbreviations

GABA: γ-aminobutyric acid
MDA: malondialdehyde
POD: peroxidase
SOD: superoxide dismutase
